# Role of Endogenous Salicylic Acid as a Hormonal Intermediate in the Bacterial Endophyte *Bacillus subtilis*-Induced Protection of Wheat Genotypes Contrasting in Drought Susceptibility under Dehydration

**DOI:** 10.3390/plants11233365

**Published:** 2022-12-03

**Authors:** Oksana Lastochkina, Sergey Ivanov, Svetlana Petrova, Darya Garshina, Alsu Lubyanova, Ruslan Yuldashev, Bulat Kuluev, Evgenia Zaikina, Dilara Maslennikova, Chulpan Allagulova, Irina Avtushenko, Albina Yakupova, Rashit Farkhutdinov

**Affiliations:** 1 Institute of Biochemistry and Genetics UFRC RAS, 71 Pr. Oktyabrya, 450054 Ufa, Russia; 2 Ufa Institute of Chemistry UFRC RAS, 69 Pr. Oktyabrya, 450054 Ufa, Russia; 3 Department of Biology, Bashkir State University, 32 Zaki Validi, 450076 Ufa, Russia

**Keywords:** endophytic *Bacillus subtilis*, *Triticum aestivum* L., salicylic acid, 1-aminobenzotriazole, drought tolerance

## Abstract

Endophytic *Bacillus subtilis* is a non-pathogenic beneficial bacterium which promotes plant growth and tolerance to abiotic stresses, including drought. However, the underlying physiological mechanisms are not well understood. In this study, the potential role that endogenous salicylic acid (SA) plays in regulating endophytic *B. subtilis*-mediated drought tolerance in wheat (*Triticum aestivum* L.) was examined. The study was conducted on genotypes with contrasting levels of intrinsic drought tolerance (drought-tolerant (DT) cv. Ekada70; drought-susceptible (DS) cv. Salavat Yulaev). It was revealed that *B. subtilis* 10-4 promoted endogenous SA accumulation and increased the relative level of transcripts of the *PR-1* gene, a marker of the SA-dependent defense pathway, but two wheat cultivars responded differently, with the highest levels exhibited in DT wheat seedlings. These had a positive correlation with the ability of strain 10-4 to effectively protect DT wheat seedlings against drought injury by decreasing osmotic and oxidative damages (i.e., proline, water holding capacity (WHC), and malondialdehyde (MDA)). However, the use of the SA biosynthesis inhibitor 1-aminobenzotriazole prevented endogenous SA accumulation under normal conditions and the maintenance of its increased level under stress as well as abolished the effects of *B. subtilis* treatment. Particularly, the suppression of strain 10-4-induced effects on proline and WHC, which are both contributing factors to dehydration tolerance, was found. Moreover, the prevention of strain 10-4-induced wheat tolerance to the adverse impacts of drought, as judged by the degree of membrane lipid peroxidation (MDA) and plant growth (length, biomass), was revealed. Thus, these data provide an argument in favor of a key role of endogenous SA as a hormone intermediate in triggering the defense responses by *B. subtilis* 10-4, which also afford the foundation for the development of the bacterial-induced tolerance of these two different wheat genotypes under dehydration.

## 1. Introduction

Wheat (*Triticum aestivum* L.) is a major cereal crop that is grown worldwide [1]. Drought stress leads to significant reductions in yield and productivity, posing a serious threat to food security [2]. As climate change increases the prevalence of drought, this threat to agriculture is exacerbated [3,4]. Drought affects wheat plants in multifaced ways. Drought can affect wheat plant productivity, seedling growth, and seedling germination. Drought can negatively affect plants due to oxidative and/or osmotic stress, water deficit, stomata closure, decreased photosynthetic activity, altered biomass partitioning, and yield [2,5]. It is urgent to find novel ways to increase drought tolerance in wheat seedlings. The early stages of wheat growth are particularly sensitive to drought, which can have a negative impact on subsequent plant yield.

Seed priming with *Bacillus subtilis*, an endophytic plant growth-promoting bacteria (PGPB), has been shown to promote plant growth and tolerance, mitigating the effect of drought stress [6,7,8,9,10]. The growth-stimulating and protective effects of *B. subtilis* under various environmental stresses have been demonstrated in many plants [11,12], including wheat [10,13,14,15]. PGPB *B. subtilis* promoted the growth and development of plants under various biotic/abiotic conditions indirectly or directly by initiating a wide range of defense responses associated with plant resistance/tolerance, thus improving crop productivity [8,9,10,16,17]. The known mechanisms behind the influence of *Bacillus* spp. on host plants include: (i) competition for space/nutrients with phytopathogenic microorganisms; (ii) improvement of macro/-micronutrient bioavailability (i.e., atmospheric N fixation, P solubilization, and siderophore production) [18,19,20,21,22]; (iii) the production of different bioactive substances (i.e., compounds with antibiotic and phytohormone-like activities) and signaling molecules; (iv) the regulation of plant hormone levels (i.e., indole-3-acetic acid, cytokinins, abscisic acid, salicylic acid (SA)) [9,10,13,23,24]; and (v) the induction of plant systemic resistance and tolerance [6,7,15,16,25,26,27]. The efficacy of microbial inoculants depends on a variety of strain-dependent factors such as the ability to produce multiple plant growth-promoting traits and the ability to colonize the surface (epiphytes) or interior (endophytes) of the plant. Endophytic *B. subtilis* strains may be more effective at protecting plant growth under long-term stress conditions because the conditions inside the plant (stable pH, humidity, nutrient flux, and a lack of competition from many microorganisms) offer a stable host environment, allowing the bacteria to thrive and manifest their positive effects [28,29]. However, the behavior of any bacterial strain is at least partially dependent on the host species or varietal characteristics, geographic origin, environment, and stress type/level during growth [30,31,32,33,34,35]. It is crucial to understand the mechanisms that underlie the interactions between host plants and endophytic *B. subtilis* under drought. This will help in the creation of a commercial inoculant that can be used to sustain wheat production under climate change.

Plants have developed mechanisms to cope with environmental stress. These mechanisms include secondary metabolite generation, osmolyte synthesis modulation, and antioxidant system activation [2]. It was previously found that the bacterial endophyte *B. subtilis* 10-4 modulates growth, physiological, and biochemical responses in different ways, depending on the intrinsic drought sensitivity of the host wheat cultivar [32]. This variation has been correlated with the bacterial capacity to increase SA production in wheat seedlings. This correlation supports a hypothesis in which endogenous SA actively mediates the *B. subtilis*-induced effects on drought-tolerant (DT) and drought-susceptible (DS) wheat genotypes. It is also established that SA is a major phenolic compound involved in plant development and growth and in the promotion of systemic acquired resistance (SAR) under a variety of biotic/abiotic stressors [36,37,38]. The correlation between SAR development and the accumulation of PR (pathogenesis-related) proteins in plants under SA influence is evidenced [39], and the *PR-1* gene is a molecular biomarker of SA-driven defense reactions [16,39]. There is also information about the sensitivity to different genes coding for PR-proteins (*PR-1* among them) in treatments with different growth regulators (SA, jasmonic acid, brassinosteroids) [40,41] and beneficial microbes [16,42]. To date, the protective effect of SA on abiotic stress tolerance is well documented in many plants [36,38]. It is less clear what mechanisms are targeted by endogenous SA under drought conditions, particularly in relation to PGPB inoculation. Recent findings demonstrate the capacity of SA to directly influence root microbiome composition by modulating the colonization process by certain bacterial taxa [43]. This could be due to the gating of bacterial taxa, as a result of SA activity, or via currently undefined effects of microbe–microbe interactions or root physiology. The root microbiomes of plants with altered SA signaling show a greater diversity than wild type plants in their bacterial populations. Different bacteria strains utilize SA in different ways: either as a growth signal or as a source of carbon [43]. For example, SA from PGPB *Pseudomonas aeruginosa* PF23EPS+ promotes sunflower growth in salt soils infected with the plant pathogen *Macrophomina phaseolina* [44]. Taken together with our understanding that bacterial inoculation and drought result in endogenous SA accumulation in wheat [13,32], these data support a model in which bacterial-induced SA and drought signaling can be linked, and the effect of bacteria on the regulation of drought tolerance depends on the level of SA. It is not clear what role endogenous SA plays in PGPB-mediated drought tolerance in wheat or by which pathway endogenous SA is produced under these conditions.

SA is synthesized in plants via two distinct pathways: isochorismate syntheticase (ICS) and phenylalanine amino-lyase (PAL) [45]. SA is synthesized in the ICS pathway from chorismic acid and catalyzed with ICS and isochorismate pyruvatelyase (IPL). IPL was originally discovered in *P. fluorescens* and *P. aeruginosa* [46], but an IPL homolog has not yet been characterized in plants. In 2019, it was shown that plants have a different ICS pathway than bacteria. In this plant-specific pathway, isochorismate is conjugated with the amino acid glutamate to produce an intermediate molecule (isochorismate-9-glutamate), which then either decomposes spontaneously or is enzymatically catalyzed into SA [45]. L-phenylalanine, which is part of the PAL pathway, is converted to transcinnamic acids by PAL. SA is then produced via two intermediates: ortho-coumaric or benzoic acids (BA) [47]. BA 2-hydroxylase (BA2H) converts BA into SA. Earlier studies have shown that the structural analog of phenylalanine, 2-aminoindane-2-phosphonic acid, is a competitively effective inhibitor of PAL in vitro and in vivo [48]. Similarly, the activity of BA2H can be inhibited by 1-aminobenzotriazole (ABT) [49].

This study was designed to assess the role of SA in regulating endophytic *B. subtilis*-induced tolerance to drought in wheat. These effects were measured in different contexts by using *Triticum aestivum* L. genotypes with diverse drought-adaptive strategies and by using ABT, an inhibitor of SA biosynthesis. To examine the role of *B. subtilis*-generated SA in regulating drought tolerance in wheat and the effect that intrinsic tolerance has on these mechanisms, the changes in endogenous SA contents in bacterial-inoculated and drought-stressed wheat seedlings in the presence of the SA biosynthesis inhibitor ABT were investigated. The effects of *B. subtilis* 10-4, when applied together with an SA inhibitor, on leaf WHC, membrane lipid peroxidation, proline, and seedling growth were also assessed.

## 2. Results

### 2.1. Endogenous SA Regulates the Pre-Adaptive Influence of B. subtilis 10-4 on Wheat Genotypes Contrasting in Drought Susceptibility under Dehydration

**Figure 1:**
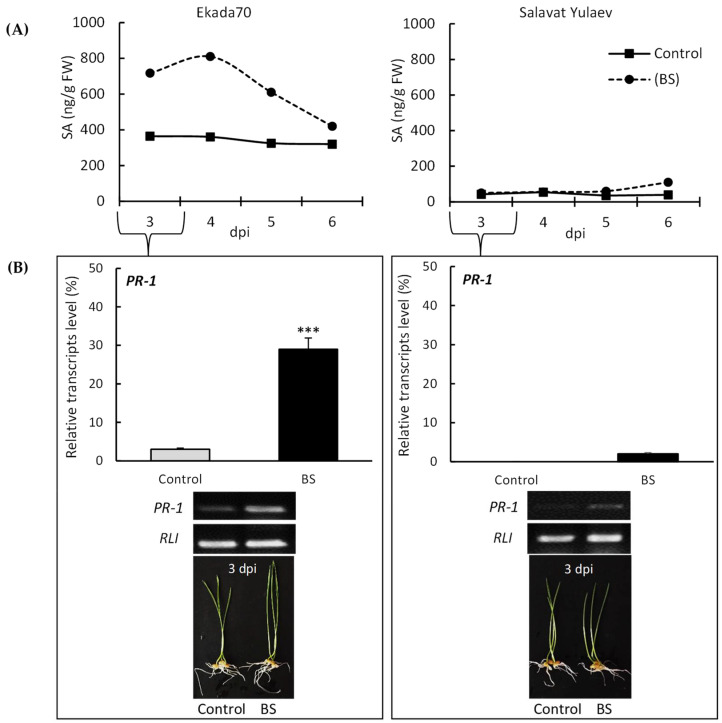
Influence of *B. subtilis* 10-4 (BS) on endogenous salicylic acid (SA) accumulation (**A**), and the relative level of PR-1 gene transcripts versus the reference gene *RLI* (RNase L inhibitor-like protein) (**B**) in seedlings of wheat contrasting in drought susceptibility (cv. Ekada70, drought-tolerant (DT); cv. Salavat Yulaev, drought-susceptible (DS)). FW—fresh weight; dpi—days post-bacterial inoculation. The average data of three independent replicates and their SEM were presented. ***—indicates a significant difference between uninoculated control and inoculated seedlings (*p* ˂ 0.05).

Our data indicate that the concentration of endogenous SA in control wheat seedlings (non-bacterized and non-stressed) differs by cultivar (Figure 1A). The highest endogenous SA concentration was found in DT cv. Ekada70, and the lowest was found in DS cv. Salavat Yulaev (Figure 1A). *B. subtilis* 10-4 led to an increase in endogenous SA in the seedlings of cv. Ekada70, peaking at 4 dpi. However, in seedlings of cv. Salavat Yulaev, different accumulation patterns were found in which the SA content was static over the first 4 dpi and became visible only after 5 dpi (Figure 1A). In response to *B. subtilis* 10-4, the concentration of endogenous SA DT cv. Ekada70 seedlings increased 1.8-fold at 3 dpi, peaked at a 2.2-fold concentration at 4 dpi, and gradually decreased to control values. In contrast, DS cv. Salavat Yulaev seedlings exhibited measurable SA accumulation at 5 dpi (1.5 times increase in SA) that increased to 2.5-fold concentrations at 6 dpi (Figure 1A). Thus, the findings suggest that both of these genotypes, in response to bacterial inoculation, accumulate endogenous SA, but in different, genotype-specific manners: the DT genotype had a quicker response; the DS genotype had a belated response.

*B. subtilis* 10-4 mediated endogenous SA levels were accompanied by an increase in the relative levels of *PR-1* gene transcripts in bacterial-inoculated seedlings (3 dpi) of the Ekada70 genotype, reaching maximum values of about 30%. In contrast, the DS cv. Salavat Yulaev seedlings did not exhibit any significant change in the transcript level (about 2%) (Figure 1B). The results are consistent with the data regarding endogenous SA variations in these seedlings. This indicated the importance of SA as an intermediate in the realization of B. subtilis 10-4 actions on wheat plants contrasting in drought susceptibility.

### 2.2. Identification of the Efficiency and Optimum of SA Biosynthesis Inhibitor Concentration

To determine the contribution of endogenous SA to the *B. subtilis* 10-4 impact on wheat plants, an inhibitory analysis using 1-aminobenzotriazole (ABT, an inhibitor of SA biosynthesis) (Sigma-Aldrich, St. Louis, MO, USA) was conducted. The effect of ABT in different concentrations on endogenous SA biosynthesis in wheat seedlings treated with endophytic strain 10-4, the growth of bacterial cells in vitro, and their capacity to colonize the inner tissues of wheat has been verified.

#### 2.2.1. Influence of the SA Biosynthesis Inhibitor on Endogenous SA in *B. subtilis*-Inoculated Wheat Genotypes Contrasting in Drought Susceptibility

**Figure 2:**
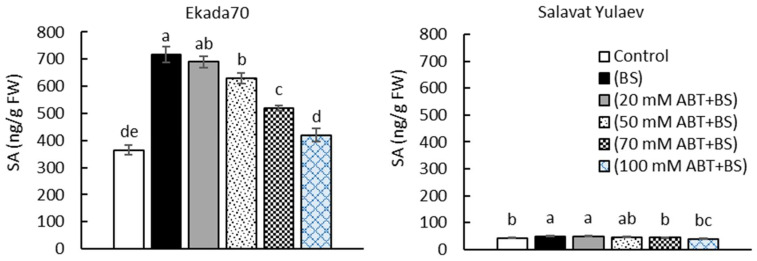
The influence of 1-aminobenzotriazole (ABT) (salicylic acid (SA) biosynthesis inhibitor) on the content of endogenous SA in *B. subtilis* 10-4 (BS)-inoculated wheat seedlings (3 dpi). The bars represent the average values of the three replicates ± SEM. Various letters show a significant difference between the averages at *p* ˂ 0.05.

The results showed that, depending on the increase in ABT concentration, the content of *B. subtilis*-induced endogenous SA decreased in 3 dpi seedlings of DT cv. Ekada70 and reached almost the control seedlings levels upon 100 mM of ABT (Figure 2). This finding suggests that *B. subtilis* 10-4 might be involved in controlling de novo SA synthesis in these seedlings. As for DS cv. Salavat Yulaev, at the same stage of ontogenesis (3 dpi), there were no significant changes in endogenous SA upon the co-application of strain 10-4 and ABT in comparison with DT cv. Ekada70.

#### 2.2.2. Influence of the SA Biosynthesis Inhibitor on the Growth of *B. subtilis* 10-4 Cells In Vitro and the Capacity Colonizing Wheat Seedlings’ Inner Tissues

The influence of ABT at a range of concentrations on the growth of *B. subtilis* 10-4 bacterial cells in vitro in Petri dishes containing LB nutrient medium was tested. It was found that *B. subtilis* 10-4 bacterial growth decreased slightly in proportion to inhibitor concentration (see Supplemental Figure S1) but was not completely suppressed. These data indicate that the penetration of bacteria into plant tissues and their colonization are not significantly affected in the presence of ABT.

**Figure 3:**
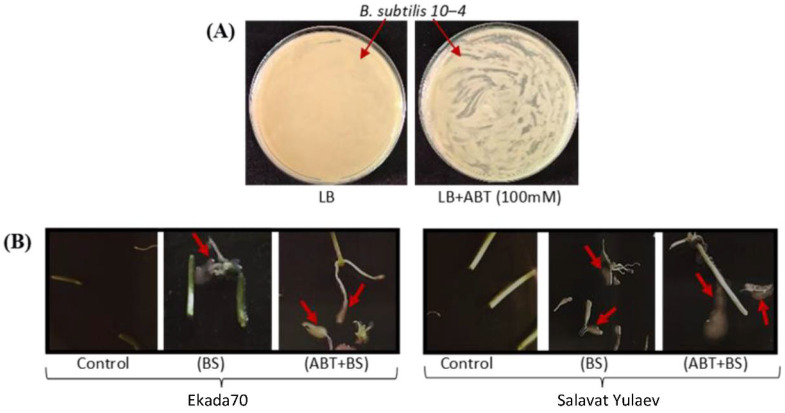
Influence of the SA biosynthesis inhibitor 1-minobenzotriazole (ABT) (100 mM) on the growth of *B. subtilis* 10-4 bacteria cells in Petri dishes with LB nutrient medium (**A**) and the capacity of *B. subtilis* 10-4 (BS) colonizing surface-sterilized wheat seedlings’ inner tissues (3 dpi) (**B**). Red arrows indicate the growth of *B. subtilis* 10-4 bacteria around the segments of surface-sterilized seedlings of wheat.

Furthermore, the influence of the treatment with 100 mM ABT was assessed, which was effective in the prevention of strain 10-4-mediated endogenous SA accumulation in seedlings (Figure 2) and regarding the capacity of strain 10-4 to colonize the inner tissues of surface-sterilized seedlings of wheat. The results showed no bacterial growth around the control (uninoculated with *B. subtilis* 10-4) wheat seedling segments. In *B. subtilis* 10-4-inoculated variants, both in the absence and presence of ABT, the growth of bacteria was observed (Figure 3B). Thus, the use of ABT (100 mM) does not prevent the penetration and growth of endophyte bacteria *B. subtilis* 10-4 in the inner tissues of seedlings of both wheat cultivars but does reduce the strain 10-4-induced endogenous SA at nearly the level of control within them. So, this concentration of ABT (100 mM) was used in further experiments.

### 2.3. An Inhibitory Analysis of Endogenous SA’s Role in the Regulation of B. subtilis 10-4 Caused Protective Effects on Wheat Seedlings under Drought

#### 2.3.1. Influence of the SA Biosynthesis Inhibitor on Endogenous SA in *B. subtilis*-Inoculated and Drought-Stressed Wheat Seedlings

**Figure 4:**
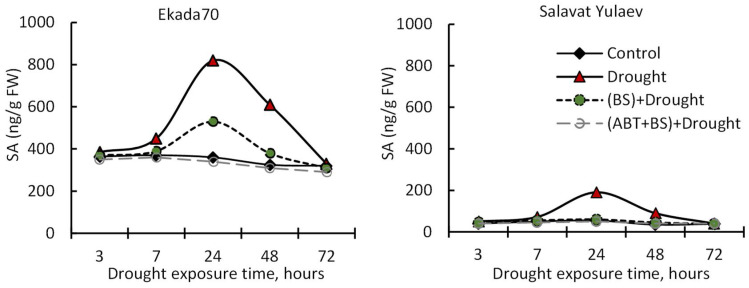
Effect of the SA biosynthesis inhibitor 1-aminobenzotriazole (ABT) (100 mM) on the endogenous salicylic acid (SA) accumulation in *B. subtilis* 10-4 (BS)-inoculated wheat seedlings under drought (12% PEG-6000). Wheat seedlings (3 dpi) were transferred in 12% PEG-6000 for 72 h.

It was discovered that incubation in 12% PEG-6000 (drought stress) caused a reversible rise in endogenous SA levels in wheat seedlings, reaching maximum amounts after 24 h of stress exposure (Figure 4) of up to two times for cv. Ekada70 and four times for DS cv. Salavat Yulaev (indicating that DS seedlings react more strongly to stress), followed by a gradual decrease to control values by 72 h of stress exposure (6-day-old seedlings). *B. subtilis* pretreated seedlings in DT cv. Ekada70 had a visibly lower level of PEG-caused SA accumulation (Figure 4A). So, under stress conditions in cv. Ekada70, the application of *B. subtilis* 10-4 helped maintain the SA level by 125% relative to the control value (Figure 4A). Inoculation with *B. subtilis* 10-4 neutralized stress-caused endogenous SA accumulation in seedlings of cv. Salavat Yulaev (Figure 4B). This is consistent with our finding of delayed SA accumulation in this cultivar (Figure 1A). However, the application of ABT completely prevented *B. subtilis*-caused endogenous SA changes in the seedlings of both cultivars under drought. The endogenous SA content in stressed seedlings treated with *B. subtilis* 10-4 and ABT was comparable to that of control ones (Figure 4).

#### 2.3.2. Influence of the SA Biosynthesis Inhibitor on *B. subtilis*-Inoculated Seed Germination, Seedling Growth, and Leaf WHC under Drought

**Figure 5:**
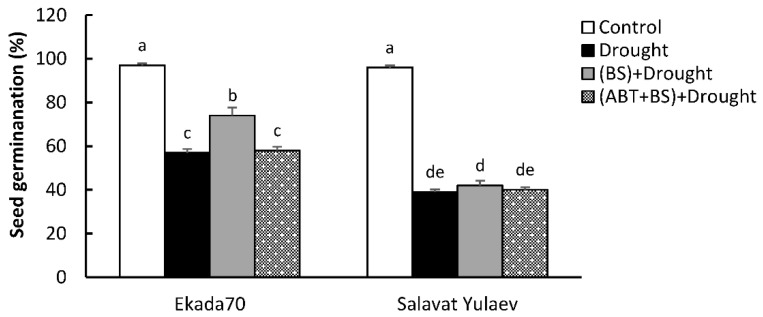
The effect of the treatment with the 1-aminobenzotriazole (ABT) SA biosynthesis inhibitor on the seed germination percentage of *B. subtilis* 10-4 (BS)-inoculated (3 dpi) cv. Ekada70 and cv. Salavat Yulaev under drought conditions (12% PEG-6000). The bars represent the average values of the three replicates ± SEM. Various letters show a significant difference between the averages at *p* ˂ 0.05.

**Figure 6:**
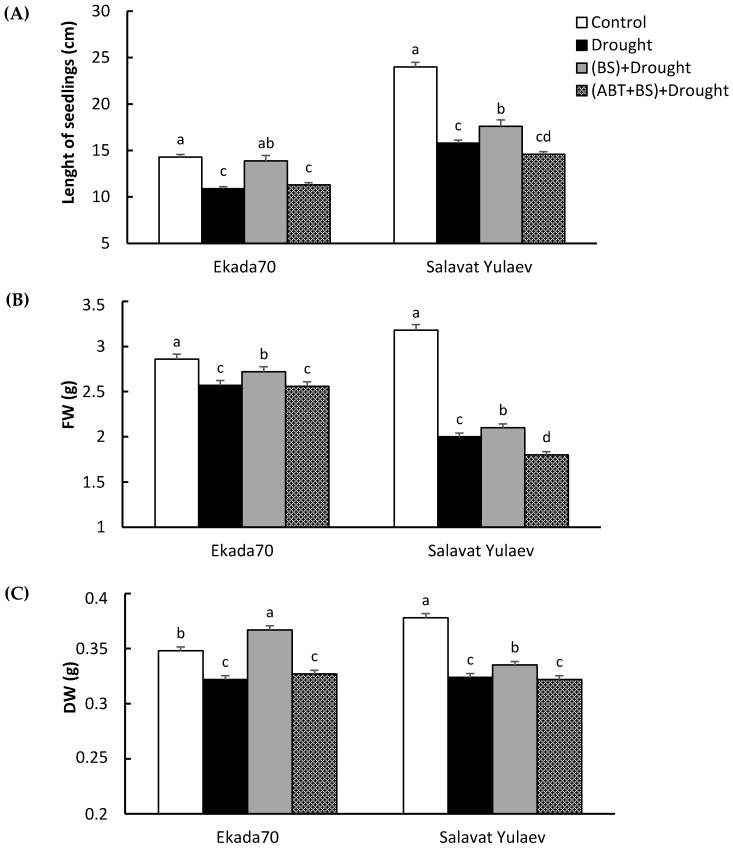
Effect of the salicylic acid biosynthesis inhibitor 1-aminobenzotriazole (ABT) and *B. subtilis* 10-4 (BS) on the growth of 6-day-old wheat seedlings (length (**A**), fresh weight (FW) (**B**), and dry weight (DW) (**C**)) under drought stress. The duration of drought stress exposure (12% PEG-6000) was 72 h. The bars represent the average values of the three replicates ± SEM. Various letters show a significant difference between the averages at *p* ˂ 0.05.

In order to evaluate the regulatory role of SA for *B. subtilis* 10-4-induced defense responses, the experiments using the SA biosynthesis inhibitor ABT in wheat plants was performed. It was effective in blocking bacterial-induced SA accumulation when applied at 100 mM (Figure 2 and Figure 4). Seed germination and the linear dimensions of non-inoculated or inoculated wheat seedlings under experimental conditions with and without ABT and under normal and drought conditions were measured. The results of this comparative analysis are shown in Figure 5 and Figure 6.

Drought significantly decreased the germination rate for DT cv. Ekada70 (by 1.7 times) and DS cv. Salavat Yulaev (by 2.5 times) (Figure 5). For DT cv. Ekada70, the application of *B. subtilis* 10-4 increased seed germination during drought by up to 1.3 times compared to an uninoculated control. Particularly, under drought, the germination was increased from 57% in non-bacterized seeds to 74% in bacterized seeds. However, the protective effect of *B. subtilis* 10-4 on the seed germination percentage upon drought was prevented by the simultaneous use of *B. subtilis* 10-4 and the endogenous SA biosynthesis inhibitor ABT and was 58%. As for DS cv. Salavat Yulaev, there was no apparent protective effect of *B. subtilis* 10-4 on seed germination under drought. Particularly, under drought, the germination in uninoculated and inoculated seeds was 39% and 42%, respectively, while in groups with the co-application of *B. subtilis* 10-4 and ABT, the seed germination was 40% (Figure 5).

Exposure to drought for 72 h resulted in a reduction in the wheat seedling length, as well as a decreased DW and FW (Figure 6). Drought has reduced the seedlings’ length by 1.3 (cv. Ekada70)–1.5 (cv. Salavat Yulaev) times (Figure 6A); FW by 1.1 (cv. Ekada70)–1.8 (cv. Salavat Yulaev) times (Figure 6B); and DW by 0.95 (cv. Ekada70)–1.45 (cv. Salavat Yulaev) times (Figure 6C). Pretreatment with *B. subtilis* 10-4 reduced the adverse effects of drought on the early wheat seedlings’ growth of both DS and DT cultivars (Figure 6). However, this protective effect was eliminated when in groups with a joint application of *B. subtilis* 10-4 and ABT; the length of seedlings was similar to that of the control. Similar results were obtained when assessing the influence of ABT on the FW and DW of bacterial-inoculated and drought-stressed wheat seedlings (Figure 6B,C).

In normal growth conditions, the co-application of *B. subtilis* 10-4 with ABT reduced the bacterial ability (after individual application) to promote wheat seedlings growth and biomass accumulation (not presented).

**Figure 7:**
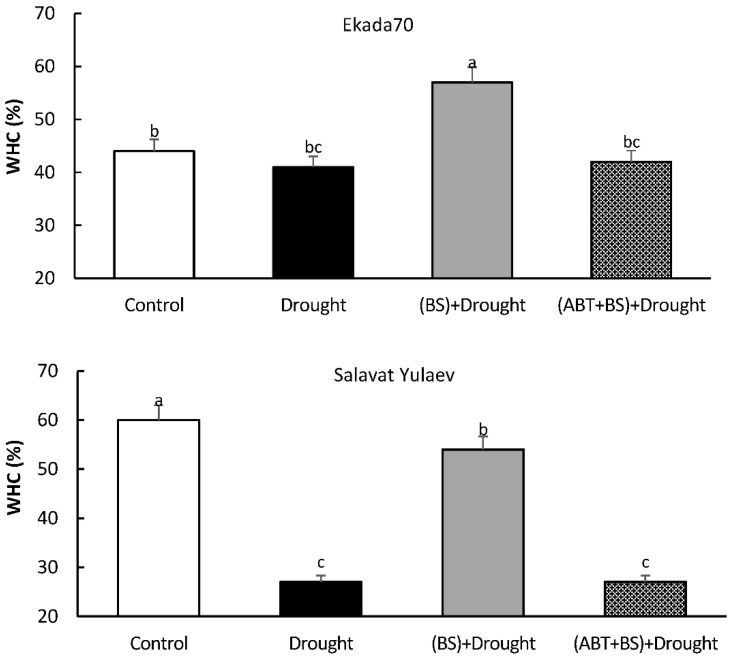
Influence of the seed treatment with the endophytic bacterium *B. subtilis* 10-4 (BS) in the presence of the salicylic acid biosynthesis inhibitor 1-aminobenzotriazole (ABT) on the leaf’s water-holding capacity (WHC) of cv. Ekada70 and cv. Salavat Yulaev wheat plants (21 days post-bacterial inoculation) under drought (12% PEG-6000). The time of drought exposure (12% PEG-6000) was 14 days. The bars represent the average values of the three replicates ± SEM. Various letters show a significant difference between the averages at *p* ˂ 0.05.

The results also revealed that inoculation with *B. subtilis* 10-4 improved (to differing degrees) WHC in both wheat cultivar leaves under drought (Figure 7). However, the co-application of *B. subtilis* 10-4 together with ABT prevented this endophyte capacity to improve leaves’ WHC under drought stress.

#### 2.3.3. Influence of SA Biosynthesis Inhibitor Application on Oxidative (MDA) and Osmotic (Proline) Damages in *B. subtilis* 10-4-Inoculated and Drought-Stressed Wheat Seedlings

**Figure 8:**
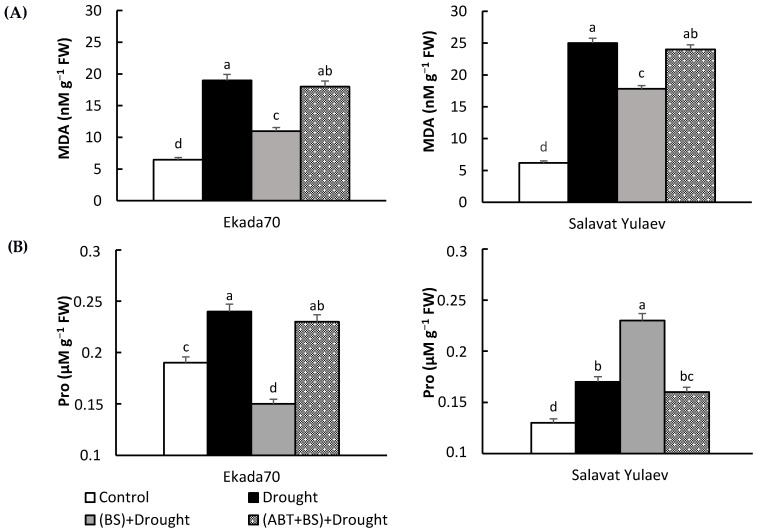
Influence of seed treatment with *B. subtilis* 10-4 (BS) in the presence of the SA biosynthesis inhibitor 1-aminobenzotriazole (ABT) on the lipid peroxidation (MDA) (**A**) and proline (Pro) concentration (**B**) in 4-day-old *Triticum aestivum* L. seedlings under drought. Control—untreated and unstressed seedlings. The duration of drought stress (12% PEG-6000) is 24 h. The bars represent the average values of the three replicates ± SEM. Various letters show a significant difference between the averages at *p* ˂ 0.05.

An exposure of 3-day-old seedlings to 12% PEG for 24 h led to a significant increase in proline and lipid peroxidation (i.e., malondialdehyde (MDA)) levels (Figure 8). *B. subtilis* (strain 10-4)-treated seedlings subjected to drought showed decreased levels of stress-induced MDA accumulation compared with the MDA levels found in non-bacterized stressed seedlings (Figure 8A). It was found that, under drought in DS and DT genotypes, upon bacterial treatment, the content of proline was changed in different manners (Figure 8B). In particular, in the bacterial-inoculated plants of DT cv. Ekada70, stress-induced proline accumulation was effectively decreased and was even lower than that in the control, while in DS cv. Salavat Yulaev, an additional proline accumulation was observed (Figure 8B). ABT completely prevented such *B. subtilis* 10-4-mediated changes in proline or MDA under drought for both genotypes. In summary, the seedlings co-treated with *B. subtilis* 10-4 and ABT under drought conditions did not differ in the content of MDA and proline from untreated control plants subjected to drought (Figure 8).

## 3. Discussion

Our results demonstrated that seed inoculation with *B. subtilis* 10-4 significantly improved the growth parameters (increased germination of seeds, elongation of seedlings, their biomass) for the drought-tolerant (DT) genotype of wheat under severe drought conditions (Figure 5 and Figure 6). These were accompanied by an increase in endogenous SA in them as a result of bacterial inoculation (Figure 4). However, for the drought-susceptible (DS) genotype, there was no apparent protective effect under drought on seed germination (Figure 5), but other growth parameters (seedlings length, biomass) were also improved but were not as good as those for DT cv. Ekada70 (Figure 6). There was also less bacterial influence on the SA level (Figure 4). It was assumed that endogenous SA might be a hormone intermediate in the realization *B. subtilis* 10-4-induced plant pre-adaptation to the forthcoming drought stress. It is known that SA is a key factor in maintaining the drought tolerance of plants [36,37,38]. The participation of SA in the *B. subtilis* 10-4-realized cascade of reactions is also confirmed by the accumulation of transcripts of the *PR-1* gene in bacterial-treated seedlings (Figure 1B). The *PR-1* gene is a biomarker of SA-driven defense reactions which plays an important role in the regulation and protection of plants against a variety of biotic and abiotic stressors [16,40,41,42,50,51,52,53,54]. There is information about sensitivity to different genes coding for PR-proteins (*PR-1* among them) in treatments with different growth regulators [40,41] including PGPB [16,42]. At once, a complex picture of the interaction of signaling pathways is observed in plants, which is indicated by the different nature of the influence of the formation of systemic tolerance by strain 10-4, depending on the plant variety and its strategy of adaptation to drought, which is manifested in a different amount of *PR-1* gene transcription (or even its absence) in DT and DS wheat genotypes. Particularly, our results demonstrated a significant increase in *PR-1* gene transcripts in seedlings from DT cv. Ekada70 as a result of strain 10-4 inoculation, which was preceded with a significant rise in their endogenous SA. The transcriptional activity of the *PR-1* gene and endogenous SA in the seedlings of DS cv. Salavat Yulaev was the lowest (Figure 1A,B). The findings can serve as indirect evidence in favor of the fundamental capacity of *B. subtilis* 10-4 to form the induced systemic tolerance of these wheat plants to drought with the involvement of the salicylate-dependent signaling pathway.

To test the role of endogenous SA as an intermediate, the experiments where wheat seedlings were treated before sowing with ABT, an inhibitor of SA biosynthesis, and *B. subtilis* 10-4 with the subsequent measurement of different indicators of cell metabolic activities were conducted. It should be noted that the SA biosynthesis inhibitor concentration (100 mM), which was selected as effective in this study (Figure 2 and Figure 4), is comparable to that used in some other recently published works [55,56,57].

The colonization of the inner tissues of the plant by endophytes plays a major role in forming effective microbial–plant interactions and is the factor influencing biological activity [8,28,58]. The finding demonstrated that the co-application of *B. subtilis* 10-4 and ABT did not prevent the bacterial capacity to colonize inner wheat tissues. This indicates that the ability of bacteria from the inside to influence metabolism is still preserved (Figure 3B). However, co-treatment with ABT significantly reduced cv. Ekada70- and completely prevented cv. Salavat Yulaev *B. subtilis*-induced SA accumulation in wheat seedlings (Figure 2) and abolished the effects of *B. subtilis* treatment on stressed plant growth (Figure 6). This indicates that *B. subtilis* may exert positive effects via regulating endogenous SA synthesis in wheat plants. The presented results expand our knowledge of the early responses of wheat plants of different genotypes to seed priming with the bacterial endophyte.

It was very important to assess how the effect of the inhibitor action was extended over time and stable. To do this, the leaf’s WHC in 21-day-old plants was evaluated. The main response of plants to drought stress is to close their stomata in order to reduce water loss through transpiration [59,60]. It was reported earlier that PGPB could regulate water content by altering hydraulic conductivity and stomatal opening in plants [61,62]. For instance, *Pseudomonas azotoformans*-treated wheat plants demonstrated a 16% rise in relative water content when compared to untreated plants under drought stress [62]. Obviously, the observed increase in WHC in leaves upon *B. subtilis* 10-4 inoculation (Figure 7) may serve as an indicator enhancing the tolerance of plants to dehydration. However, the application of ABT (an SA biosynthesis inhibitor) completely prevented the capacity of *B. subtilis* 10-4 to increase WHC, suggesting the prevention bacterial-induced wheat tolerance under drought. By increasing the leaf WHC, plants can reduce their direct and indirect water costs. It spreads water uptake across the day, buffers peak demand, and allows them to take up water when it is less expensive to do so, such as when salinities are low, when temperatures are cooler with a higher relative humidity, during rain events, or overnight when the stomata are closed [63,64].

PGPB is capable of synthesizing osmoprotectants and increases their concentrations in plants. This helps to facilitate osmotic adjusting mechanisms, protect plants against stress-induced osmotic damages, and support water status [8,65,66,67,68,69,70]. *Streptomyces pactumAct12*, for example, was found to increase the tolerance and growth of wheat plants that are subject to PEG-induced drought [71]. Similar results were obtained by Sandhya and colleagues [72]. *Pseudomonas putida* GAP-P45 also increased proline accumulation, increased relative water content, and maintained maize plant cell water levels during drought [72]. For these bacterial-inoculated plants, there was an increase in gene expression and the accumulation of osmoprotectants (i.e., proline, glutamine, and betaine), as well as the activation of various mechanisms responsible for rapid upregulation signaling pathways [71,72]. Our results also demonstrated the ability of *B. subtilis* 10-4 to regulate proline metabolism—but differently in DS and DT wheat genotypes during drought (Figure 8B)—and both were effective. This is evidenced by the decreased damage caused by drought to the integrity of membrane structures (Figure 8A) and WHC, which was tested on wheat ontogenesis for 21 days (Figure 7). This indicates a prolonged protective effect of *B. subtilis* on these wheat genotypes. Obviously, *B. subtilis* caused changes in proline, along with the role in osmoregulation, which can also protect the structure of various biomolecules and membranes [67,68,73]. In addition, it can act as scavenger of free radicals that protect DNA against the harmful effects of reactive oxygen species (ROS) [67,68,74,75]. However, the co-application of *B. subtilis* 10-4 with ABT completely prevented the changes in proline observed after sole bacterial application under the same drought conditions (Figure 8B). This suggests that *B. subtilis* 10-4-caused endogenous SA contributes to the osmotic adjustment of bacterial-treated plants under drought.

Today, many studies have shown that PGPB can improve different plant stress tolerances through the modulation of the components of pro- and antioxidant systems and decreasing stress-caused oxidative damages of the cell membranes [15,25,26,76,77,78,79,80,81]. Our data are also consistent with the literature and show the capacity of *B. subtilis* 10-4 to decrease drought-induced lipid peroxidation in wheat plants of both genotypes (Figure 8A). This indicates a significant role for antioxidant system regulated by strain 10-4 in order to protect plants. However, co-application with ABT completely prevented such protective effect of bacteria on cell membranes against drought-caused oxidative damages. This suggests that *B. subtilis* 10-4-induced endogenous SA plays a crucial role in the induction of plant defense mechanisms, leading to decreased lipid peroxidation in bacterial-inoculated plants under drought.

## 4. Materials and Methods

### 4.1. Plant Materials and Bacterial Strain

The experiments were conducted in hydroponically grown wheat (*Triticum aestivum* L., drought-tolerant (DT) cv. Ekada70, and drought-sensitive (DS) cv. Salavat Yulaev) plants. The seeds were supplied by the Chishminsky Breeding Station of the Bashkir Research Institute of Agriculture UFRC RAS (Chishmy, Russia). The endophytic bacterial strain *B. subtilis* 10-4 was previously isolated from arable soils. It was identified using 16S rRNA [13], characterized in detail [13,32], and registered in the Russian National Collection of Industrial Microorganisms (VKPM) (number B-12988).

### 4.2. Inoculum Preparation and Seed Treatment

*B. subtilis* 10-4 cells were cultivated in Lauria–Bertani (LB) medium for 24 h at 37 °C and 180 rpm until the cell concentration reached 10^8^ CFU mL^−1^ [81,82]. To obtain the inoculum, the suspension was diluted down to 10^5^ CFU mL^−1^ using sterile H_2_O and was monitored by measuring the optical density at 600 nm (SmartSpecTM Plus spectrophotometer, Bio-Rad, Hercules, CA, USA).

### 4.3. Design of Experiments and Growth Conditions

Seeds were sterilized in 96% ethanol for 1 min and washed with sterile H_2_O for 2–3 min. Thereafter, depending on the analysis, the surface-sterilized seeds were: 

(1) immerged in *B. subtilis* 10-4 (10^5^ CFU mL^−1^) solution or sterile H_2_O (control) for 1 h and grown hydroponically in Petri dishes with H_2_O to measure the *PR-1* gene transcription level (in 3 days) and endogenous SA content (in 3, 4, 5, 6 days) (Figure 1);

(2) immerged in *B. subtilis* 10-4 (10^5^ CFU mL^−1^) solution, sterile H_2_O (control), ABT (20, 50, 70, 100 mM), and *B. subtilis* 10-4 (10^5^ CFU mL^−1^) + ABT (20, 50, 70, 100 mM) for 1 h. The treated seeds were further grown hydroponically in Petri dishes with ABT (20, 50, 70, 100 mM) solutions or H_2_O (control) for three days, followed by endogenous SA assessment (Figure 2);

(3) immerged in *B. subtilis* 10-4 (10^5^ CFU mL^−1^) solution, sterile H_2_O (control), ABT (100 mM), and *B. subtilis* 10-4 (10^5^ CFU mL^−1^)+ABT (100 mM) for 1 h. The treated seeds were grown hydroponically in Petri dishes with H_2_O (control), ABT (100 mM), or solutions of 12% PEG-6000 or 12% PEG-6000+ABT (100 mM) for 3 days, followed by seed germination % assessment (Figure 5);

(4) immerged in *B. subtilis* 10-4 (10^5^ CFU mL^−1^) solution, sterile H_2_O (control), ABT (100 mM), and *B. subtilis* 10-4 (10^5^ CFU mL^−1^) + ABT (100 mM) for 1 h. The treated seeds were grown hydroponically in Petri dishes with sterile H_2_O or ABT (100 mM) for 3 days. Afterwards, the seedlings were moved to glasses with the same solutions and grew in the same conditions. Samples of the plant (roots, shoots, or whole seedlings) were collected after 3, 7, 24, 48, and 72 h of stress exposure to evaluate physiological and biochemical parameters (i.e., endogenous SA (Figure 4), growth (Figure 6), proline, MDA (Figure 8)). To measure the leaf WHC, 6-day-old seedlings that grew under normal conditions were further exposure to stress (12% PEG-6000) for 14 days (21 days after seed sowing (Figure 7).

In all cases, the seeds and seedlings were hydroponically growth under a long photoperiod of the day (16 h light/8 h dark, 200 μmoL m^−2^ s^−1^) at 21–24 °C.

The growth parameters were assessed (germination %, seedlings length, FW, and DW) by the classical methods [83].

### 4.4. Determination of Water Holding Capacity (WHC)

The leaf WHC has been tested as outlined by Shchukin and Gromov [84]. Briefly, three fresh 21-day-old wheat seedling leaves were cut, weighed, and held for 3–4 h under 50% RH and 25 °C. Thereafter, these leaves were weighed and then dried at 105 °C. WHC has been calculated as a percent of total water content [84]. In each variant, three leaves in four replicates was used (*n* = 3, 4 replicates).

### 4.5. Assessment of B. subtilis 10-4 Cells’ Capacity to Grow In Vitro in the Presence of ABT in the Growth Medium

*B. subtilis* 10-4 cells’ capacity to grow in vitro in the presence of ABT was studied by plating the bacterial strain 10-4 suspension (10^8^ CFU mL^−1^) in Petri dishes with LB solid medium prepared with the addition of different concentrations of ABT (0–100 mM, 10 mM increments) (tests) or without ABT addition (control). Thereafter, the dishes were cultivated at 37 °C for 24 h. This experiment was carried out in three replicates. Visualization of the growth of bacterial cells in LB Petry dishes (with ABT) was performed using a Canon PowerShot SX540 HS digital camera (20.3 Mpix Zoom50x 3 ′′1080).

### 4.6. Assessing the Capacity of B. subtilis 10-4 in the Presence of 1-Aminobenzotriazole (ABT) to Colonize Inner Wheat Plant Tissues

The capacity of the bacterial strain 10-4 to colonize inner plant tissues in the presence of the SA biosynthesis inhibitor ABT was determined using surface-sterilized wheat seedlings (3 days old) pretreated with *B. subtilis* 10-4 and *B. subtilis* 10-4 + ABT. The leaf and root segments were immersed in 70% ethanol for 5 min. Afterwards, the ethanol was drained, and the segments were washed with sterile H_2_O for three times with subsequent air drying for 10–15 min. The surface-sterilized segments were placed in Petri dishes with LB medium and kept for 72 h at 28 °C for bacterial growth. Clean cultures of isolates from surface-sterilized seedling segments were analyzed for the identity of the original strain 10-4 using random amplified polymorphic DNA analysis by polymerase chain reaction (RAPD-PCR) [13]. The DNA of the bacteria was isolated by applying lysis buffer (1% Tryptone100, 1% Tween20, 1% Chelex100, 0.005% Cresol Red, dH_2_O). The genetic polymorphism of the strain was assessed based on the RAPD-PCR results of total DNA using AFK primers (50-gcgtccattc-30). Amplification was carried out using Tercik equipment (DNA Technology, Moscow, Russia). The analysis and visualization of the RAPD results were carried out using horizontal electrophoresis in 1.5% agarose gel in an electrophoretic chamber SE-2 (Helikon, Moscow, Russia) at 25 kV for 1 h. The gel was stained with EtBr, and the results were recorded using the Gel Doc XR system (Bio-Rad, Hercules, CA, USA).

### 4.7. Endogenous Salicylic Acid (SA) Assay

The total SA was analyzed by high-performance liquid chromatography (HPLC) [85]. The plant tissue (seedling) (0.2–0.3 g) was extracted using 20 mL of dH_2_O (90–100 °C) and incubated at 100 °C for 30 min with subsequent cooling. Membrane filters (0.45 µm) (Chromafil Xtra PTFE–45/13, Macherey-Nagel GmbH Co, Duren, Germany) were used to filter the extracts. The analysis was caried out by a Waters Breeze chromatograph (Waters Corporation, Milford, MA, USA) with a Waters 2487 Dual & Absorbance diode array detector at 305 nm. A 250 × 4.6 mm Pursuit C18 5 µm column (Agilent Technologies, Santa Clara, CA, USA) was used. As a mobile phase, 0.5% solution of H_3_PO_4_: acetonitrile = 65:35 (1.0 mL min^−1^) was used. A total of 20 µL of the extract was introduced into the chromatography system using an automated sampler Waters 2707 (Waters Corporation, Milford, MA, USA). The software calibration curve was used in calculating the total SA content.

### 4.8. Analysis of the Relative Level of PR-1 Gene Transcripts

The total RNA was isolated from wheat seedlings using a Trizol in accordance with the protocol (Thermo Fisher Scientific Inc., Waltham, MA, USA). The concentration of the isolated RNA (previously dissolved in sterile mQH_2_O) was measured at A260/A280 by a SmartSpecTM Plus spectrophotometer (Bio-Rad, Hercules, CA, USA). Contaminated genomic DNA was removed from RNAs with DNAse (Thermo Fisher Scientific Inc., Waltham, MA, USA) before cDNA synthesis. To obtain cDNA based on the RNA of the studied samples, a reverse transcription reaction was performed using a reverse transcriptase according to the protocol (Sintol, Moscow, Russia). The obtained cDNA was applied to the amplification. PCR was performed in an amplifier, TP4 PCR01 Tercik (DNA Technology, Moscow, Russia), with a primer 5’ cacctattagctagctaatcat 3’ (F) and 5’ gtacgtactgtacgtaacatatgta 3’ (R) for *Triticum aestivum PR-1* gene sequences [86]. Electrophoresis for the separation of PCR products was performed in a 7% polyacrylamide gel using a MiniProteanII Electrophoretic CtII (Bio-Rad, USA). To visualize the amplification products, the gel was incubated in a solution of ethidium bromide (0.5 µg mL^−1^) for 10 min. Thereafter, it was viewed in a transilluminator Gel Doc XR (Bio-Rad, Hercules, CA, USA) and photodocumented using a Gel Camera System (Bio-Rad, Hercules, CA, USA). The obtained data were processed using the computer programs LabWorks 4.6 and TotalLab, v. 2.01 (UVP, Inc., Upland, CA, USA). To determine the size of the amplicons, the marker DNA GeneRulerTM 100 bp DNA Ladder (Fermentas, Vilnius, Latvia) was used. The transcription activity data of the studied *PR-1* gene were normalized against the transcriptional activity of the *RLI* gene and are presented in arbitrary units [87].

### 4.9. Lipid Peroxidation Assay

The degree of lipid peroxidation of membranes was assessed by malondialdehyde (MDA) concentration [83]. Fresh leaf (0.5 g) was homogenate with dH_2_O (3 mL), with the subsequent addition of 20% trichloroacetic acid (3 mL), followed by centrifugation for 10 min at 10,000× *g*. Afterword, the supernatant (2 mL) was blended with 0.5% thiobarbituric acid (2 mL), heated (100 °C for 30 min), and then rapidly cooled. The optic density of the obtained solution was measured at 532 nm and 600 nm (SmartSpec^TM^ Plus spectrophotometer, Bio-Rad, St. Louis, MO, USA). Using an extinction coefficient (155,000 L cm^−1^ moL^−1^), the concentration of MDA (nmoL g^−1^ FW) was calculated.

### 4.10. Proline Determination

The proline concentration was determined in accordance with Bates et al. [88]. Fresh seedlings (0.5 g) were placed in the tubes with boiled water (2.5 mL) and incubated for 30 min in a water bath (100 °C). Thereafter, they were taken off and cooled. This extract (1 mL) was mixed with a solution of ninhydrin acid (1 mL) and glacial acetic acid (1 mL). Thereafter, the samples have been incubated in a water bath (100 °C) for 1 h and cooled using an ice vessel. The optic density of the resulting solutions was measured at 522 nm using a spectrophotometer SmartSpecTM Plus (Bio-Rad, St. Louis, MO, USA). A calibration curve was used to calculate the proline concentration (mg g^−1^ FW).

### 4.11. Statistical Analysis

All physiological, biochemical, and molecular analyses were carried out in three biological and three analytical replicates. The results represented the average values of the three replicates as the mean ± standard error (SEM). Statistically significant differences between the mean values were estimated using an analysis of variance (ANOVA), followed by the Tukey test (*p* < 0.05).

## 5. Conclusions

Thus, through the treatment of DT and DS wheat cultivars with *B. subtilis*, we found that *B. subtilis* inoculation increased SA accumulation, but two wheat cultivars responded differently, with the highest levels exhibited in DT wheat seedlings. The addition of ABT, an inhibitor of SA biosynthesis, decreased SA and abolished the effects of *B. subtilis* treatment. The findings demonstrate a major role of endogenous SA accumulation and maintaining its elevated level in bacterial pretreated seedlings under drought stress in implementing the pre-adaptive and protective effects of *B. subtilis* 10-4 on wheat plants, respectively. The results presented here established that using ABT prevented *B. subtilis* 10-4-caused endogenous SA elevation under normal conditions and the maintenance of its increased level under stress. This was accompanied by the prevention of the capacity of *B. subtilis* 10-4 to protect wheat seedlings against drought injury by decreasing its cell’s osmotic and oxidative damages (i.e., proline, WHC, and lipid peroxidation (MDA)) and plant growth (length, biomass). The findings provide an argument in favor of the realization of endogenous SA as a hormonal intermediate in the manifestation of the anti-stress effect of *B. subtilis* 10-4 on these two different wheat genotypes under drought stress. In addition, this testifies to the support of the development of plant systemic tolerance upon *B. subtilis* 10-4’s application with the involvement of the SA-dependent signaling pathway. This study provided experimental evidence to reveal the mechanisms of *B. subtilis*’s beneficial wheat growth under drought stress.

## Supplementary Materials

The following supporting information can be downloaded at: https://www.mdpi.com/article/10.3390/plants11233365/s1, Figure S1: Influence of salicylic acid (SA) biosynthesis inhibitor 1-aminobenzotriazole (ABT) in a range of concentrations (0–100 mM) on the growth of bacteria *B. subtilis* 10-4 in Petry dishes with LB nutrient medium.
