# Supplementary material for: Role of Endogenous Salicylic Acid as a Hormonal Intermediate in the Bacterial Endophyte Bacillus subtilis-Induced Protection of Wheat Genotypes Contrasting in Drought Susceptibility under Dehydration
Source: Plants (Basel). 2022 Dec 3;11(23):3365. doi: 10.3390/plants11233365 (PMC9736644; doi:10.3390/plants11233365)
Supplement: Supplementary file 1 [file plants-11-03365-s001.zip › plants-2012296-supplementary.pdf]

Supplementary Figure S1.

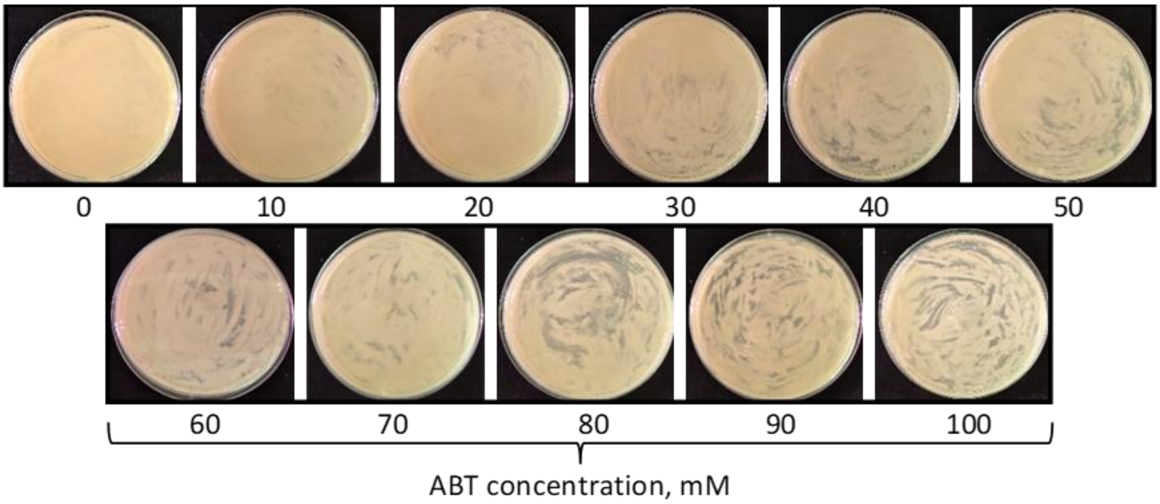

Figure S1. Influence of salicylic acid (SA) biosynthesis inhibitor 1-aminobenzotriazole (ABT) in range of concentrations (0-100 mM) on the growth of bacteria *B. subtilis* 10-4 in Petry dishes with LB nutrient medium.
